# Current results on the biological and pharmacological activities of Indole-3-carbinol

**DOI:** 10.17179/excli2017-1028

**Published:** 2018-02-06

**Authors:** Jae Kwang Kim, Sang Un Park

**Affiliations:** 1 Division of Life Sciences and Convergence Research Center for Insect Vectors, Incheon National University, Incheon 22012, Korea; 2 Department of Crop Science, Chungnam National University, 99 Daehak-ro, Yuseong-gu, Daejeon, 34134, Korea

## ⁯

Dear Editor,

Indole-3-carbinol (I3C; C_9_H_9_NO) is a phytochemical that is derived from the breakdown of the glucosinolate, glucobrassicin. I3C is present at relatively high levels in most cruciferous vegetables such as broccoli, cabbage, cauliflower, brussels sprouts, collard greens, and kale (Fujioka et al., 2016[14]; Licznerska and Baer-Dubowska, 2016[22]). The enzyme, myrosinase (β-thioglucosidase), catalyzes the hydrolysis of glucosinolates in intact plant cells (Zhao et al., 2015[37]). After chopping or chewing of raw cruciferous vegetables, the plant cells are damaged and glucobrassicin is exposed to myrosinase. This catalyzes the conversion of glucobrassicin to a glucose molecule and an unstable aglycone, which is hydrolyzed to thiohydroximate-O-sulfonate (de Vos et al., 2008[9]). If the sulfate ion is released spontaneously, this may form another unstable intermediate, 3-indolylmethylisothiocyanate. This released compound readily converts to a thiocyanate ion and I3C (Kim et al., 2008[20]). 

I3C has recently become available as a nutritional supplement and it provides an attractive natural product for drug development in the pharmaceutical industry. It has been reported to show diverse promising biological properties, with anti-atherogenic, antioxidant, anti-carcinogenic, and anti-inflammatory activities (Fuentes et al., 2015[13]; Maruthanila et al., 2014[25]). I3C has attracted considerable attention in recent years within the pharmaceutical and functional food industries. Here, we summarize recent studies performed to evaluate the biological and pharmacological activities of I3C (Table 1(Tab. 1); References in Table 1: Mohammadi et al., 2017[27]; Ampofo et al., 2017[1]; Hammerschmidt-Kamper et al., 2017[16]; Safa et al., 2017[31]; Kabel et al., 2017[19]; Sherer et al., 2017[33]; Gehrcke et al., 2017[15]; Quirit et al., 2017[30]; Fletcher et al., 2017[12]; Julliard et al., 2017[18]; Kundu et al., 2017[21]; Wang et al., 2016[35]; Megna et al., 2016[26]; El-Naga and Mahran, 2016[10]; Enríquez et al., 2016[11]; Poindexter et al., 2016[29]; Song et al., 2015[34]; Lin et al., 2015[23]; Busbee et al., 2015[4]; Safa et al., 2015[32]; Wang et al., 2015[36]; Caruso et al., 2014[6]; Perez-Chacon et al., 2014[28]; Aronchik et al., 2014[2]; Chen et al., 2014[7]; Choi et al., 2014[8]; Mao et al., 2014[24]; Jayakumar et al., 2014[17]; Brandt et al., 2014[3]; Busbee et al., 2014[5]). 

## Acknowledgements

This research was supported by Golden Seed Project (213006051WTE11) funded by the Ministry of Agriculture, Food and Rural Affairs (MAFRA), Ministry of Oceans and Fisheries (MOF), Rural Development Administration (RDA) and Korea Forest Service (KFS), Republic of Korea.

## Conflict of interest

The authors declare no conflict of interest.

## Figures and Tables

**Table 1:**
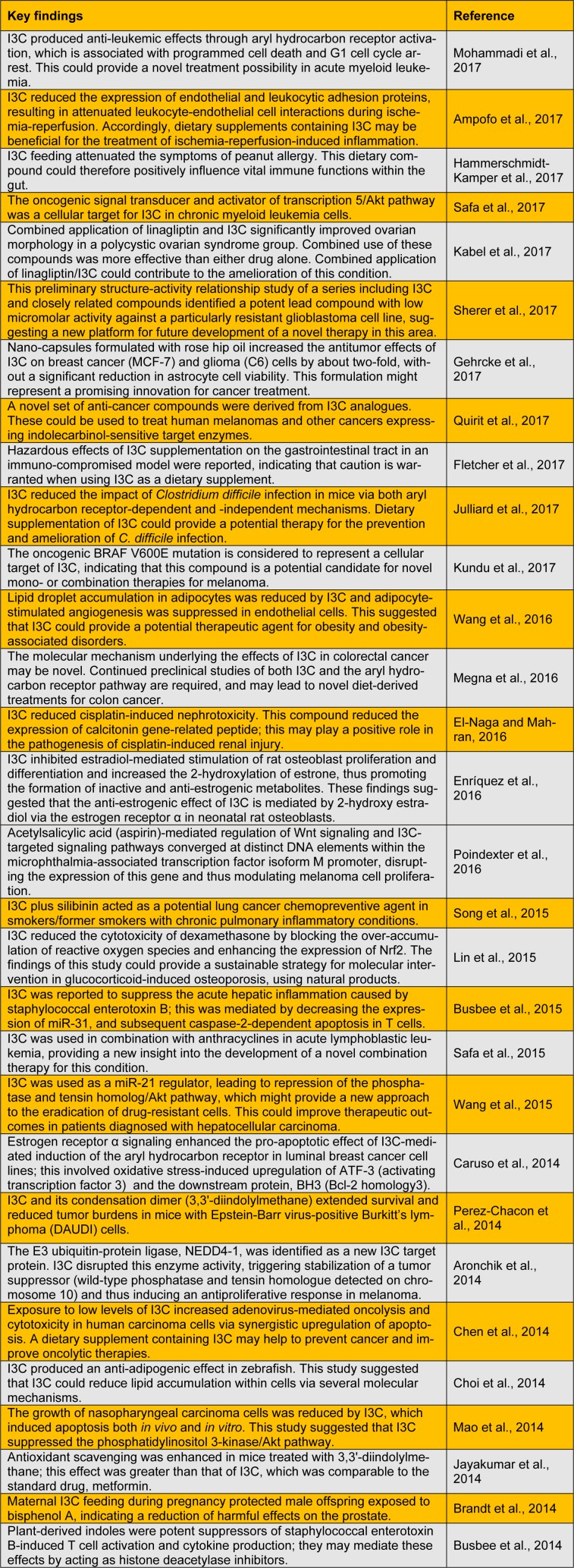
Recent studies on the biological and pharmacological activities of Indole-3-carbinol
